# Special Issue: “Peter Biely, A Pioneering Researcher in the Enzymology of Plant Biomass Degradation”

**DOI:** 10.3390/molecules26164857

**Published:** 2021-08-11

**Authors:** Maria Hrmova

**Affiliations:** 1 School of Agriculture, Food and Wine, Faculty of Sciences, University of Adelaide, Glen Osmond, SA 5064, Australia; maria.hrmova@adelaide.edu.au; Tel.: +61-8-8313-0755; 2 School of Life Science, Huaiyin Normal University, Huai’an 223300, China

As it has been outlined on the website of the Special Issue entitled “Peter Biely, a pioneering researcher in the enzymology of plant biomass degradation” in the journal Molecules (section Macromolecular Chemistry, ISSN 1420-3049), plant biomass is a key renewable resource. Although it encompasses a valuable energy resource, the economic feasibility of its bioconversion to biofuels, biochemicals, and other high-value products is challenging. This is due to insufficient knowledge of the composition and structure of plant cell walls, and their biosynthesis and biodegradation, which make the cell wall structure–function relationships incompletely tractable. However, an extraordinary increase in the rate of accumulation of knowledge has been made in the last 10 years. The plant cell wall complexity at the general and special levels, due to multifaceted interconnections amongst carbohydrates, proteins, organic molecules, ions, and other components, creates that essential physical barrier for individual plant cells to protect themselves from surrounding environments and microorganisms. Nonetheless, it is this pressure from the latter that has led to the formation of resilient plant cell wall structures during evolution, and which constantly evolve. Nevertheless, scientists have been addressing these challenging processes of plant cell walls and biomass degradation and uncovered a wide range of microbial biocatalysts (enzymes) that harness energy stemming from covalent linkages contained in plant cell walls.

This issue focuses on carbohydrate-active enzymes involved in the biodegradation of plant cell walls and biomass, with an emphasis on the mechanisms of their biodegradation. As inferred from above, the Special Issue explicitly examines plant cell wall-degrading enzymes, their mode of action, substrate binding, molecular mechanisms, and catalytic principles that underlie the biodegradation of plant cell walls. From a practical point of view, this uncovering of the fundamentals of enzymes operating during plant biomass degradation aims to contribute to progress towards the bioconversion of plant biomass. This knowledge, based on its deconstruction, seeks to obtain biofuels, create high-quality foods from plant biomass, and be applied in general to the pulp and paper industries.

In the light of the outlined focus, we invite articles from all fields of the enzymology of plant cell walls and biomass degradation, whether they are short or full-length research papers, review articles, opinions, commentaries, or other formats. We are looking forward to these new experimental and theoretical contributions that would add to further progress in the field and create opportunities for established and novice scientists, professors, and students to publish their research and share ideas.

This Special Issue serves as a tribute to biochemist RNDr. Peter Biely, DrSc., a chief scientist at the Institute of Chemistry of the Slovak Academy of Sciences Commitment and loyalty to science, limitless enthusiasm, and passion for making discoveries in the field of the enzymology of plant biomass degradation: these are the hallmarks of the career of RNDr. Peter Biely, DrSc, who marks his 80th birthday.

Peter was born into a teacher’s family in a small village in Slovakia and equipped with perseverance and responsibility to accomplish. After he completed his university undergraduate degree in analytical chemistry, he joined the Institute of Chemistry of the Slovak Academy of Sciences. Here, under the guidance of Dr. Štefan Bauer, Peter studied metabolic transformations of deoxy-sugars and received his PhD diploma in carbohydrate biochemistry. In 1968, Peter, on his first international appointment with Professor Roger Jeanloz at the Harvard Medical School, worked on the isolation of sugar nucleotides. After his return from the US, Peter continued working on deoxy-sugars and the mechanism of resistance to catabolic repression in yeasts exposed to 2-deoxy-d-glucose and 2-deoxy-2-fluoro-d-glucose.

In 1974, Peter, with Zdeněk Krátký, Mária Vršanská, and Mária Cziszárová, the members of his group at the Institute of Chemistry, embarked on a long and productive journey of the bioconversion of plant biomass to produce renewable energy resources and biochemicals. This work coincided with the 1970 oil crisis. Peter, with his group, collaborated with Dr. Anna Kocková-Kratochvílová, the founder and curator of the Yeast Culture Collection at the Institute of Chemistry, on the biodegradation of xylan, the underrated plant cell wall component, to isolate xylanolytic organisms, amongst them *Cryptococcus* and *Aureobasidium* strains. This research led to the isolation of xylanolytic enzymes: secreted endo-β-1,4-d-xylanase, and intracellular β-d-xylosidase [1]. The detailed characterisation of one of the enzymes—endo-β-1,4-d-xylanase—was published in several back-to-back, highly cited articles in 1980–1981 in the, at that time, highly influential European Journal of Biochemistry (now known as FEBS Journal) [2], which was a remarkable feat. Notably, in one of these and other papers, Peter introduced to endo- and exo-acting glycanases the ‘subsite-mapping’ formalism that has been since in usage. The works on the xylanolytic system of *Cryptococcus* become highly cited—at the time of this writing, these outputs received around 450 citations.

Later, Peter became interested in other xylanolytic (with Ilona V. Gorbacheva, Moscow), and xylanolytic and cellulolytic systems (with Eva Petráková and Maria Hrmova) and published a series of articles in the Archives of Microbiology and other journals with over 350 citations. Then came the collaboration with the late Oskar Markovič and Danica Mislovičová that led to the development of covalently modified soluble chromogenic polysaccharides (xylans dyed with Remazol Brilliant Blue and hydroxyethylcellulose dyed with Ostazine Brilliant Red) and their applications in the identification of a variety of endo-acting hydrolases on electrophoretic zymograms. This research directed works published in Analytical Biochemistry [3] and Methods in Enzymology (jointly with over 600 citations) and led to further work on non-specific endo-β-1,4-glucanases (cellulases) hydrolysing xylan and defining the mode of action of cellobiohydrolases (300 citations). Currently, the selection of polysaccharide substrates based on similar chemistry is available from the Neogen (previously known as Megazyme) and Merck-Life Science companies. From these works, the review of microbial xylanolytic enzyme systems followed and represents one of the most cited works from Peter [4]; this, with his inter-laboratory testing of methods for the xylanase activity assay [5], jointly aggregated nearly 4000 citations. 

In 1984, Peter, as a visiting researcher, joined the National Research Council of Canada (Ottawa) with Henry Schneider and worked on the bioconversion of xylose to ethanol. Here, Peter learned about plant xylans being acetylated. Later, based on this single piece of knowledge, he launches a new chapter in his career. Discoveries of numerous de-esterifying enzymes followed, such as acetyl xylan esterases [6], feruloyl esterases, glucuronoyl esterases [7], acetyl esterases, and α-glucuronidase [8]. With the collaborators at the Center for Agricultural Utilization Research in Peoria, Peter identified homologous genes encoding glucuronoyl esterases in then available sequenced genomes, and with a glucuronoyl esterase, researchers explored details of kinetics and substrate specificity. Notably, some of these esterases are now classified in newly established families of the Carbohydrate-Active enZYmes database (known as CAZy)—4-*O*-methyl-glucuronoyl methylesterases in the carbohydrate esterase family 15 and acetyl esterases in the carbohydrate esterase family 16; these discoveries opened a new door for lignocellulose biodegradation. The work on the breakdown of complex plant xylan structures has been summarised in previous [9] and current [10] reviews.

The originality of Peter’s research prompted invitations to lecture and to contribute with high profile review articles to leading journals, and to guide young scientists and students around the world serving as a guest scientist or as Visiting Professor. Peter, during his international engagements, worked in numerous countries, to name a few: the US (working on lignin biodegradation; endo-β-1,4-d-mannanases used for bleaching of softwood pulp; utilisation of lignocellulosic byproducts and conversion of saccharose; microbial enzymes synthesizing cycloalternan; and CE15 and CE16 transacetylases), Canada (production of endoxylanases from *Streptomyces lividans*), Japan (*Pichia stipitis* α-glucuronidase partial amino acid sequence; xylan bioconversion to prebiotic oligosaccharides), South Africa (hemicellulolytic α-glucuronidases), and Australia (barley exohydrolytic enzymes involved in mixed-linkage glucan and mannan-hydrolase enzyme systems). Some of the collaborative works with these laboratories were published in high-tier journals, for example, in the Journal of Biological Chemistry. Peter has also fulfilled various international appointments as a Visiting Professor in Chile, Brazil, Japan, New Zealand, South Africa, and Sweden.

The originality of work in Peter’s group yielded participation in nine projects funded by the European Commission programs (Copernicus, INCO Copernicus, EC RAIR, and six COST Actions) and two projects funded by NATO. Peter’s team also held collaborative agreements with Novozymes A/S in Denmark (2009–2024), bene-pharmaChem GmbH Co.K.G in Germany (2015–2017), the Norwegian University of Life Sciences in Aas (2012–2013), Annikki in Austria (2010–2011), and the Agricultural Research Service of the US Department of Agriculture in Peoria (2002–2007). The key objectives of these projects were to improve the efficacy of microbial enzymes during the saccharification of plant cell walls, through the discovery and bioengineering of novel enzymes and the introduction of efficient analytical methods to the field, and to deepen the knowledge of the fine structure of plant cell walls.

According to Web of Science, which provides access to scientific databases and citation indexing services, Peter has co-authored 230 original works from which around 30 are reviews. Peter also co-authored 31 patents. The Google Scholar search engine lists his h- and i10-indices, currently at 57 and 181, respectively, with overall citations over 14,400.

As the result of Peter’s decades-long service to the field of plant biomass degradation, in 2003 he became the Scientist of the Year of the Slovak Republic, in 2006 he was awarded the František Patočka Medal from the Czechoslovak Society for Microbiology, in 2011 he received the Order of Ľudovít Štúr by the President of the Slovak Republic, in 2015 he was bestowed the Charles D. Scott Award by the American Society for Microbiology, and he is also the recipient of the Gold Medal of the Slovak Academy of Sciences by the Vice-President of the Academy, which is its highest honour.

Due to Peter’s encyclopaedic knowledge of general and applied biochemistry, he has served in Academic Commissions for over 30 years and fulfilled important duties in numerous Committees for awarding PhD and Doctor Scientiarum degrees in the Slovak Republic and abroad. He raised numerous PhD students and delivered over 120 lectures at domestic and international venues. Peter also served ten years as the secretary and another ten years as the president of the Yeast Commission of the Czechoslovak Society for Microbiology. He co-organised numerous domestic and international meetings of the society in the picturesque Smolenice Castle and other venues and represented the Slovak Republic in the International Commission on Yeasts. These efforts have earned Peter well-deserved respect and recognition around the world.

At a personal level, I met Peter for the first time at the Smolenice Castle Yeast Conference, as an undergraduate biochemistry student, and appreciated his constructive entrées during discussions on enzymology topics. Peter is also fondly remembered by many for quick wit and humour, sometimes wry and coming out of nowhere. Last, but not least, Peter is branded as an accomplished piano player—so, we wish Peter many happy returns with music, and that he could find the largest community halls to play in, as he did in the Julia Farr Centre in Adelaide (Australia).

In summary, Peter, as a research pioneer, has made an enormous contribution to the field of xylan-degrading enzymes, carbohydrate esterases, and other enzyme systems to advance the knowledge of their catalytic function. Peter’s efforts have been directed primarily to unravel biochemical mechanisms fundamental to life. However, he also spurred significant developments in biotechnologies to inspire the development of new understanding. The knowledge that he and his colleagues and collaborators acquired is now impacting the bioengineering of novel enzymes involved in plant cell wall degradation.

The octogenarian anniversary welcomes Peter in full strength and with an ever-present appetite for new ideas. He never stops, nor does his enthusiasm and fascination with science. We wish Peter many more years to come to indulge in science, enjoy a family (and an extended family of his fellow scientists and friends), and be in good health to explore many more mechanisms that we need to know about that would enlighten our understanding of biodegradation of plant cell walls.
